# Novel electronic biosensor for automated inoculum preparation to accelerate antimicrobial susceptibility testing

**DOI:** 10.1038/s41598-021-90830-2

**Published:** 2021-05-31

**Authors:** Suzanne Putney, Andrew H. Theiss, Nitin K. Rajan, Eszter Deak, Creighton Buie, Yvonne Ngo, Hima Shah, Victoria Yuan, Elizabeth Botbol-Ponte, Adrian Hoyos-Urias, Oren Knopfmacher, Catherine A. Hogan, Niaz Banaei, Meike S. Herget

**Affiliations:** 1 Avails Medical Inc., 1455 Adams Drive, Menlo Park, CA 94025 USA; 2 Stanford University School of Medicine, 3375 Hillview Ave, Palo Alto, CA 94304 USA

**Keywords:** Biomedical engineering, Infectious-disease diagnostics

## Abstract

A key predictor of morbidity and mortality for patients with a bloodstream infection is time to appropriate antimicrobial therapy. Accelerating antimicrobial susceptibility testing from positive blood cultures is therefore key to improving patient outcomes, yet traditional laboratory approaches can require 2–4 days for actionable results. The eQUANT—a novel instrument utilizing electrical biosensors—produces a standardized inoculum equivalent to a 0.5 McFarland directly from positive blood cultures. This proof-of-concept study demonstrates that eQUANT inocula prepared from clinically significant species of *Enterobacterales* were comparable to 0.5 McF inocula generated from bacterial colonies in both CFU/ml concentration and performance in antimicrobial susceptibility testing, with ≥ 95% essential and categorical agreement for VITEK2 and disk diffusion. The eQUANT, combined with a rapid, direct from positive blood culture identification technique, can allow the clinical laboratory to begin antimicrobial susceptibility testing using a standardized inoculum approximately 2–3 h after a blood culture flags positive. This has the potential to improve clinical practice by accelerating conventional antimicrobial susceptibility testing and the resulting targeted antibiotic therapy.

## Introduction

Bloodstream infections (BSI) are diagnosed in at least 1.7 million patients in the United States each year, resulting in nearly 270,000 deaths annually^1,2^. It is well documented that timely administration of appropriate antibiotics is paramount in preventing mortality and decreasing health care costs^3–6^. Gram-negative bacteria such as *Escherichia coli*, recently identified as the most frequently isolated BSI pathogen between 2005 and 2016 following a 20 year worldwide surveillance study^7^, can harbor multiple mechanisms of resistance. This limits the utility of directly detecting specific resistance genes in positive blood cultures (PBCs) for the purposes of guiding antimicrobial therapy^8^. The ability to quickly and accurately provide the required phenotypic antimicrobial susceptibility testing (AST) data from PBCs remains challenging for the clinical laboratory^9–11^. Recently described approaches include the culture independent platform Accelerate Pheno (Accelerate Diagnostics, USA)^12^ and the dRAST (QuantaMatrix, South Korea)^13^. The novel eQUANT method described here focuses on the affordable and expedited preparation of a standardized inoculum, thereby enabling clinical laboratories to retain their legacy (0.5 McF based) AST systems while still benefiting from a faster AST result turnaround time.

Culture dependent AST requires a carefully controlled inoculum to ensure reliable results^14^. Traditionally, a PBC is subcultured on an agar plate and incubated until there is sufficient growth to make a standardized 0.5 McFarland (McF) suspension from distinct colonies, which may require 18–24 h of incubation time^8^. A 0.5 McF is a measurement of turbidity which results in a bacterial suspension equal to 1–2 × 10^8^ colony forming units per mL (CFU/mL) for *E. coli* ATCC 25922^15^. This 0.5 McF is then used directly in AST methods such as disk diffusion or is diluted further to achieve the appropriate starting inoculum for the most common automated AST platforms such as VITEK2 (BioMérieux, Marcy-l'Étoile, France), BD Phoenix (Becton Dickinson, Maryland) and MicroScan WalkAway (Beckman Coulter, California, USA).

Measurements to determine McF cannot be performed directly from PBCs due to the interference of blood cells in the optical measurement. One challenge of direct from PBC AST therefore is generating a bacterial starting inoculum at an appropriate concentration to achieve reliable AST results. Recent preliminary data from the CLSI Methods Development and Standardization Working Group indicates the potential accuracy of a direct from positive blood culture disk diffusion protocol, which is however currently limited to a select number of antimicrobials^11^.

PBCs are complex solutions which, in addition to blood and organisms, contain growth media and antimicrobial neutralizing systems with the potential to interfere with direct from PBC automated AST^11,16,17^. As a result, there have been many efforts over the years to develop methods to generate standardized 0.5 McF suspensions directly from a PBC using serum separator tubes, lysis filtration, centrifugation or simple dilutions^4,16,18–22^. While their performance is often high, the methods remain varied, manual and time-consuming and may require complex in house verification. The fact that they have not thus far been widely adopted highlights how difficult they are to implement in a clinical lab^11^. Their appeal however is clear, in that they allow a laboratory to use the AST system already in place and still reduce the timeline between a blood culture flagging positive and the initiation of AST.

Similarly, the real-time electronic sensor described here monitors bacterial growth without being affected by the blood cells present in a PBC and generates a bacterial suspension equivalent to a traditional 0.5 McF from a positive blood culture in 1–2 h. The sensor consists of a redox sensitive electrode which responds to changes in the oxidation–reduction potential (ORP) of the medium specific to microorganism metabolism. The real-time aspect of the sensor enables us to correlate changes in the ORP signal to changes in the bacterial concentration and indirectly to McF units.

**Figure 1:**
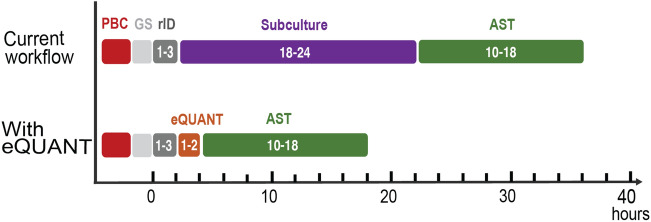
Accelerated AST workflow with eQUANT. The current laboratory workflow starts with a positive blood culture (PBC, red box) followed by a Gram stain (GS, light grey box) and rapid organism identification if available (rID, dark grey box). Bacteria from the PBC are subcultured and a 0.5 McF is prepared from colonies. The 0.5 McF is then used to lawn an agar plate for disk diffusion or utilized in an automated AST platform. On average, AST results are available in 10–18 h. With the eQUANT method, the need for the subculture step is eliminated, and the eQUANT inocula is directly generated from the PBC as soon as the organism is identified.

Over the past decade, advances in genetic and proteomic techniques have created a new paradigm for organism identification (ID) in the clinical laboratory^10^. The relatively recent introduction of matrix-assisted laser desorption ionization time-of-flight mass spectrometry (MALDI-TOF) technology into the clinical microbiology laboratory has revolutionized the identification of microorganisms and is significantly faster than traditional biochemical methods. Several laboratories have reported utilizing MALDI-TOF with direct from PBC protocols which can generate an ID result within one to two hours of a blood culture bottle flagging positive^9,23,24^. Other currently available rapid ID platforms include VERIGENE (Luminex, Austin, TX), BioFire (BioMérieux) and ePlex System (GenMark Dx). Coupling a rapid ID technique with the eQUANT method described here could allow the clinical laboratory to begin AST testing on a standardized inoculum from a PBC approximately 2–3 h after it flags positive, thereby reducing the diagnostic timeline (Fig. 1).

In this paper, we first describe the development of the eQUANT technology—the sensor, the methods, and the algorithms used to prepare a standard inoculum for use in AST. We then present the results from our initial proof-of-concept studies with direct comparisons to traditional AST approaches, as well as trials to confirm our technology’s compatibility with multiple clinical workflows.

## Results

### Sensing principle

**Figure 2:**
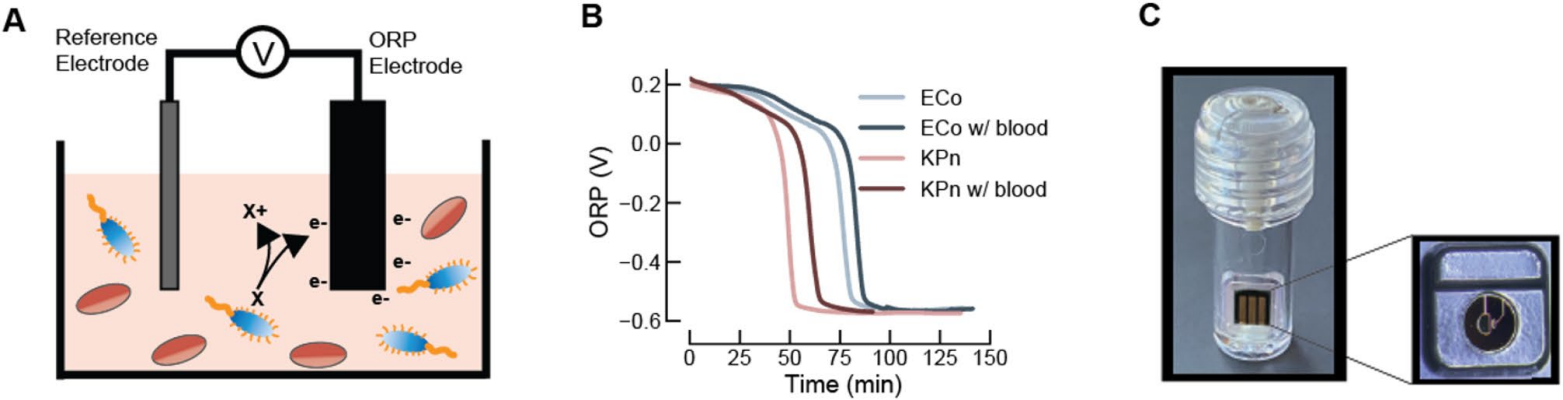
Electrical sensor principle. (**A**) The electrical sensor is composed of a sensing oxidation–reduction-potential (ORP) electrode (black) and a reference electrode (grey). The output of the sensor is the voltage difference (V) between the reference electrode and the ORP electrode. During bacterial metabolism, very reduced molecules are produced (X > X +), which readily give up electrons (e-) to the ORP sensitive sensor material. While the reference electrode maintains a fixed voltage, the bacteria change the solution ORP as electrons accumulate on the ORP sensitive sensor surface. Consequently, the overall voltage becomes more negative. (**B**) Representative ORP growth curves for two Gram-negative organisms, *E. coli* (ECo) and *K. pneumoniae* (KPn), measured in both the absence and presence of blood (PBC diluted 1:30 in growth media). (**C**) Photo of the eTube consumable with the packaged sensor integrated into the wall of the tube and contact pads facing the outside for interfacing with the read-out electronics. The inner-facing side of the sensor is shown (enlarged) to the right with a molded open cavity (black circle) exposing the ORP sensing electrode to the liquid tested.

The eQUANT sensing principle is based on measuring the changes in ORP of growth media during bacterial growth. The sensor consists of an indicating electrode which is redox sensitive and a reference electrode with constant potential. The voltage between the indicator and reference electrodes is continuously monitored as bacteria grow in a liquid culture (e.g. a diluted PBC) as shown in Fig. 2A. During growth, bacteria produce strongly reducing molecules that lower the ORP of the solution^25–27^. Reducing molecules readily give up electrons to the indicating electrode, causing the latter to accumulate negative charge. Since the reference electrode is maintained at a fixed voltage, the measured voltage (i.e. the voltage difference between indicator and reference electrodes) becomes more negative (Fig. 2B).

For use in the eQUANT instrument, the sensor is packaged and assembled into a polycarbonate cuvette—the eTube (Fig. 2C). The sensing electrodes were fabricated on a silicon wafer using common microfabrication techniques and the singulated sensor dies were wire-bonded to a 5 × 5 mm printed circuit board. This was followed by overmolding to hermetically seal the wire bonds and bond pads while leaving an open cavity, exposing the ORP sensing electrode to the solution tested.

To verify that our sensor ORP signals are not affected by the presence of blood in our growth media, and thus demonstrate viability as a method for testing directly from positive blood cultures, we measured bacterial ORP growth curves with and without blood present in the sample. A strain of *E. coli* and *K. pneumoniae* were inoculated into blood culture bottles with and without blood (1 ml dilute bacterial suspension with either 9 ml saline or blood) and grown overnight. After flagging positive, the PBC was diluted 1:30 in Mueller–Hinton growth media and incubated at 37 °C while our sensors monitored ORP signal during growth. As shown in Fig. 2B, the ORP signals during growth were not affected by the presence of blood for both species tested. Small deviations in these signals were attributed to differences in initial bacterial concentrations, resulting in small time shifts of the curves, while the characteristic shape and dynamic range remained consistent.

### Algorithm development and feasibility

**Figure 3:**
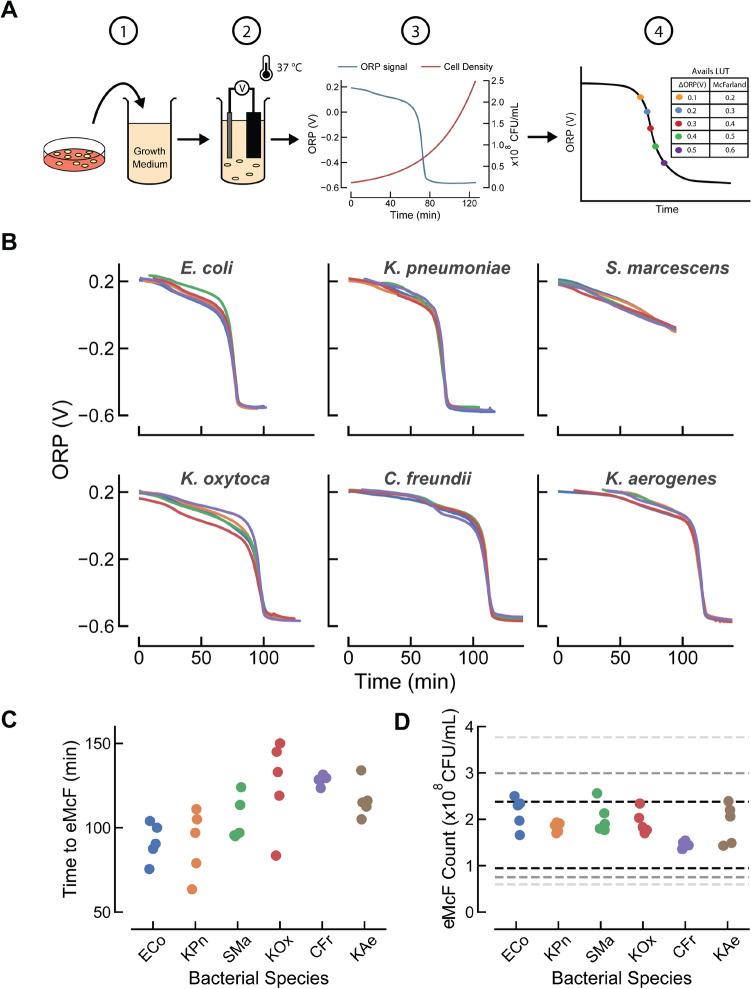
Development of eQUANT algorithms and evaluating performance. (**A**) Schematic of the look-up table generation process for a single strain, starting with bacterial colonies from a blood agar plate. Briefly, (1) colonies from the plate are suspended in growth medium at a concentration of ~ 1 × 10^7^ CFU/mL. (2) The ORP sensor is introduced to the sample which is incubated at 37 °C. (3) During growth, both ORP signal (blue) and growth (red) are measured. (4) Growth and ORP data are collected for many strains of a bacterial species. These results are combined in order to associate ORP signal (ΔORP) with a cell density (e.g. McF). Thus, a species-specific LUT is created to allow for estimation of cell density from an ORP signal. (**B**) Five different strains of six organisms (*E. coli*,* K. pneumoniae*,* S. marcescens*,* K. oxytoca*,* C. freundii*,* K. aerogenes*) were cultured and bacterial suspensions of ~ 1 × 10^7^ CFU/mL were prepared. These were grown at 37 °C and the ORP curves are shown here. (**C**) Species-specific algorithms were applied to the ORP signal curves to determine the predicted time at which an equivalent 0.5 McF was reached (time to eMcF). This was plotted as a function of the bacterial species and ranged from 64 min (one strain of *K. pneumoniae*) to 150 min (one strain of K. oxytoca). The variability in time to eMcF is due to different starting concentrations of bacteria as well as different growth rates for each strain. (**D**) The eMcF plated colony count data is plotted for all six bacterial species tested. These counts demonstrate a tight control of eMcF cell density as 28 of 30 eMcFs fell within ± 0.2log of our target concentration (1.5 × 10^8^ CFU/ml).

In addition to confirming viability of ORP measurement in the presence of blood, we also needed to determine how the real-time bacterial growth ORP signal correlates to bacterial cell density to be able to determine when samples have reached a targeted concentration. The process of building this correlation between sensor signal and cell density for a species of bacteria is shown in Fig. 3A.

**Figure 4:**
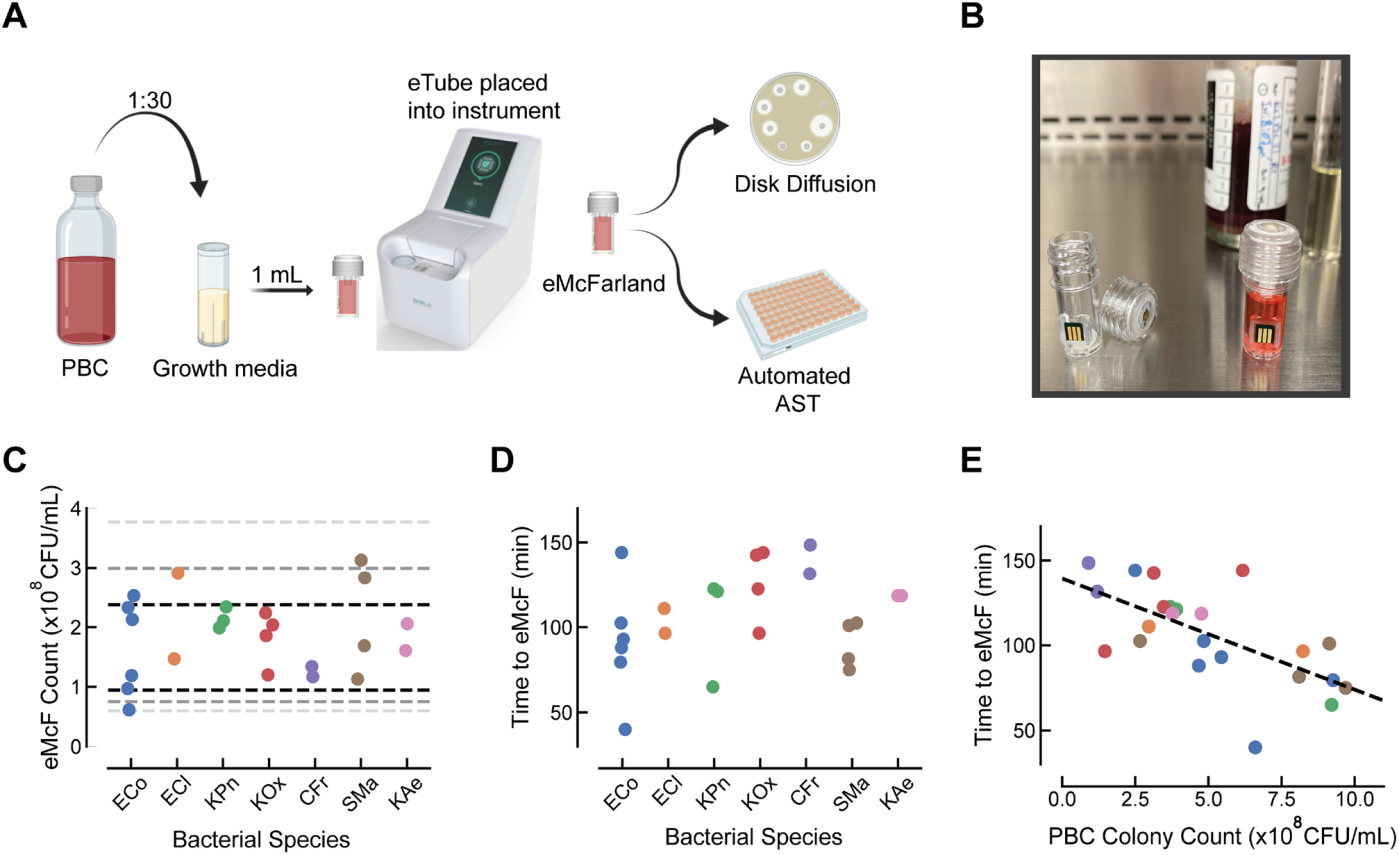
Automated eQUANT workflow and performance. The eQUANT method & instrument were used for automated eMcF generation from 23 positive blood cultures spiked with seven different species—*E. coli* (ECo), *E. cloacae* (ECl), *K. pneumoniae* (KPn), *K. oxytoca* (KOx), *C. freundii* (CFr), *S. marcescen*s (SMa), and *K. aerogenes* (KAe). (**A**) Illustration of the eQUANT workflow. An aliquot of a PBC is diluted 1:30 in growth media, transferred into the eTube and inserted into the eQUANT instrument. The organism ID is selected from the menu, and the species-specific algorithm is used for eMcF generation. The resulting standardized inoculum is then used for inoculation of conventional AST (e.g. automated AST platforms, Kirby-Bauer Disk Diffusion). (**B**) Photo of the eTube consumable with integrated ORP sensor, unfilled with cap removed (left) and filled with a diluted positive blood culture sample (right). (**C**) The resulting eMcF samples were plated and colonies were counted to determine the bacterial concentration. The dotted lines represent the error ranges of the targeted 1.5 × 10^8^ CFU/mL concentration for all species tested (± 0.2log, ± 0.3log, ± 0.4log). (**D**) Time to eMcF for all samples were recorded and ranged from 40 to 149 min with an average time of 106 ± 28 min. (**e**) Time to eMcF was plotted as a function of PBC colony counts for each species, and a Pearson Correlation was performed (dotted line). The resulting Pearson coefficient of -0.65 indicates a moderate negative correlation between time to eMcF and PBC starting concentration.

Briefly, bacterial suspensions were prepared to a concentration of approximately 1 × 10^7^ CFU/ml (roughly equivalent to 0.05 McF) in Mueller–Hinton broth using fresh colonies from a blood agar plate (Fig. 3A-1). A benchtop eQUANT equivalent setup was used to incubate samples at 37 °C using a dry bath heating block, and an ORP probe was used to monitor the real-time ORP signal throughout growth (Fig. 3A-2). Concurrent with ORP measurement, bacterial growth was measured by withdrawing samples and measuring cell density at multiple time points. These discrete values were then fitted to an exponential curve to provide a continuous growth curve that could be overlaid with the ORP signal. Figure 3A-3 depicts an example plot of an *E. coli* measurement with both the ORP signal (blue) and cell density (red) curve shown together. Using this approach, we were able to construct a strain-specific look-up table (LUT) of values by linking the change in the ORP signal (ΔORP) at various points on the sensor signal curve to a cell density value (e.g. McF). This process was repeated using multiple strains of the same bacterial species and results were then averaged to produce a single species-specific LUT (Fig. 3A-4).

This method made it possible to develop LUTs for many clinically significant Gram-negative organisms of interest and allows for real-time estimation of cell density in a growing bacterial sample using only our sensor’s ORP signal. This correlation between ORP and cell density was used to develop species-specific algorithms which determine the time point at which the bacterial concentration reaches the equivalent of a 0.5 McF. A 0.5 McF is equal to approximately 1–2 × 10^8^ CFU/ml for *E. coli* ATCC 25922^15^, thus we established the midpoint of 1.5 × 10^8^ CFU/ml as the target cell density for our eMcF (we refer to a sample grown according to the eQUANT method as an ‘eMcF’). Additional details on the types of algorithms used for these predictions as well as specific examples, are available in the Supplementary Information (Figure S1–S3).

While the work presented here describes algorithms developed and applied at the species level, the same approach of LUT generation can be expanded to encompass the genus level as well. This can be accomplished by simply expanding the strains included in the LUT generation step to include other species within the same genus. This adaptability is advantageous as the level of ID (genus or species) that can be ascertained from a sample varies between different ID methods (e.g. MALDI-TOF, VERIGENE, BioFire, or ePlex). For example, the method used by many labs may only be able to identify that an organism is from the *Citrobacter* genus and does not define a species. In this case, an algorithm developed specifically for *Citrobacter freundii* (as presented below) may not be appropriate. Instead, an algorithm can be developed that incorporates strains from multiple Citrobacter species—for example, *C. freundii*, *C. brakii*, and *C. koseri*—that would meet the need of specific ID system limitations. Thus, the eQUANT method is flexible to the myriad of requirements that exist in modern clinical labs, and unique algorithm solutions can be developed to provide eMcFs for a wide range of ID capabilities.

In order to verify the feasibility of producing an eMcF at the target concentration using our real-time prediction algorithms, new strains (i.e. strains not used in LUT, algorithm development) of each organism were spiked into blood culture bottles along with blood and grown overnight until they flagged positive. Once positive, the samples were diluted into growth media, incubated at 37 °C, and ORP sensors monitored their growth. Species-specific eQUANT algorithms were applied to the ORP signals produced during growth in order to calculate the time to eMcF. The samples were then removed at the eMcF time point and the eMcF cell density was determined by diluting and plating for colony counts (CFU/ml). The results of these experiments are summarized in Fig. 3.

The ORP signals for all strains are shown in Fig. 3B. Note that these curves have been aligned at a specific signal delta (e.g. Δ600mV, calculated from the value at 10 min) in order to account for varying initial concentrations and growth times and to more easily compare their characteristic signal shape (original ORP curves available in Supplementary Information, Figure S4). With the exception of *S. marcescens*, which drops by ~ 300 mV in total, all other *Enterobacterales* show a total amplitude of approximately 800 mV. From these plots, we observe that strains within a species exhibit exceedingly similar signals, making it possible to establish species-specific algorithms that are applicable to different strains within that species.

The time to eMcFs for the 30 strains tested (6 organisms, 5 strains each) ranged from ~ 1 to 2.5 h (avg = 110 ± 21 min, min = 64, max = 150), a substantial improvement when compared to growing colonies on an agar plate for 18–24 h (Fig. 3C). Differences in time to eMcF can be attributed to different bacterial starting concentrations (following dilution from PBC) as well as varying growth rates for the different species and strains.

The accuracy of our eMcF suspensions were evaluated using plated colony counts to compare our eMcF cell density to the target of 1.5 × 10^8^ CFU/mL (equivalent to a 0.5 McF). Figure 3D shows the bacterial concentrations of the 30 eMcFs generated using ORP sensors and our eQUANT algorithms, with dashed lines indicating ranges of 1.5 × 10^8^ CFU/mL ± 0.2log (black dashed lines), ± 0.3log (1st grey dashed lines), and ± 0.4log (2nd grey dashed lines). Our eQUANT algorithms demonstrated high accuracy in achieving target concentrations, with 28 eMcFs (93%) resulting in a bacterial concentration within ± 0.2log of our target, and all 30 (100%) within ± 0.3log.

### Preliminary trial and performance of eQUANT method

The previous section described our efforts to establish that the sensor can accurately detect ORP signal in the presence of blood and that species-specific algorithms can be implemented to grow bacterial samples to a desired concentration. A preliminary trial was subsequently undertaken to verify the accuracy of the full eQUANT method—employing the prototype eQUANT instrument, eTubes, and algorithms to prepare an eMcF from a PBC. This trial aimed to evaluate the performance of the eQUANT method (Fig. 4A) using 23 clinically significant Gram-negative organisms. Blood cultures were spiked with *E. coli* (6), *E. cloacae* (2), *K. pneumoniae* (3), *K. oxytoca* (4), *C. freundii* (2), *S. marcescens* (4), and *K. aerogenes* (2), and the eQUANT method was used to generate an AST-ready inoculum directly from the PBC.

Algorithms for each species were loaded into the instrument software, and as the sensor signal is recorded and analyzed during bacterial growth, the algorithm determines when the target cell density (1.5 × 10^8^ CFU/ml) has been reached. At that point, the instrument notifies the user and the eMcF sample is retrieved for quantification (cell density determined by diluting and plating for colony counts) and downstream antibiotic susceptibility testing. The results of these experiments were evaluated according to three criteria—time required to prepare the sample (time to eMcF), bacterial concentration of the sample (eMcF count), and the performance of the eMcF sample in AST compared to the traditional method. The eMcF time and count data is shown below in Fig. 4, and the AST results will be discussed in the following section.

The eMcF counts for these samples are plotted in Fig. 4C. Dotted lines on the graph indicate ± log ranges around the target concentration of 1.5 × 10^8^ CFU/ml. The first set of dotted lines (black) indicate a range of ± 0.2log, the next set (grey) indicate ± 0.3log, and the final set indicate ± 0.4log. Of the 23 samples tested using the eQUANT, 18 (78%) fell within ± 0.2log of our target, a narrow range that spans ~ 9.5 × 10^7^ to 2.4 × 10^8^ CFU/ml. Expanding slightly to a window of ± 0.3log, 21 (91%) successfully met this range, with one sample falling below (*E. coli* strain, eMcF count = 6.14 × 10^7^ CFU/ml) and one above (*S. marcescens* strain, eMcF count = 3.13 × 10^8^ CFU/ml) this range. All 23 samples were within ± 0.4log of our target.

The time to eMcF for these samples is plotted in Fig. 4D. The growth times varied from 40 min (*E. coli* strain) to 149 min (*C. freundii* strain). The average time required to prepare an eMcF was 106 min, with a standard deviation of 28 min. According to our initial goal of 60–120 min to generate an eMcF, our initial study was successful for 61% of samples (14 of 23). An additional three samples fall just outside of this range with a time to eMcF of 121–123 min. No samples required more than 2.5 h. The *E. coli* strain that exhibited the 40 min eMcF time is the same strain that exhibited the lowest eMcF count.

Differences in the eMcF times are hypothesized to be attributed to both variance in growth rates between individual strains as well as the significant variability in initial sample bacterial concentrations from the PBCs. We therefore analyzed if the time to eMcF correlates with the PBC concentration and performed a Pearson correlation resulting in a correlation factor of -0.65, as shown in Fig. 4E. This Pearson correlation value, which ranges from − 1 to + 1 and measures linear correlation of two variables, suggests an appreciable negative association between the cell density in a PBC and the time required to reach an eMcF, which supports what would logically be expected—that a higher initial concentration should require a shorter growth time to reach a target concentration.

### Evaluating the performance of the eMcF in AST

The performance of the eQUANT method was compared to the traditional 0.5 McF method and evaluated using both the automated VITEK2 platform as well as manual Kirby-Bauer disk diffusion. In total, 296 minimum inhibitory concentrations (MICs) from the VITEK2 and 224 zone sizes on disk diffusion were evaluated. Overall performance of the eQUANT method in AST showed essential agreement (EA) and categorical agreement (CA) of 96.6% and 95.3% respectively for VITEK2 (Table 1), and CA of 94.6% for disk diffusion (Table 2). The minor discrepancy (mD) rate was 4.4% using the VITEK2 and 4.9% for disk diffusion. The major discrepancy (MD) rate was 0.5% and 0.7% for the VITEK2 and disk diffusion, respectively. There were no very major discrepancies (VMD) observed using either method.

**Table 1:** VITEK2 GN81 antimicrobial susceptibility results for the eMcF compared to the traditional 0.5 McF.

Antibiotic (GN81 VITEK2 Card)	# isolates	# R	# S	# I	# in EA	# in CA	# mD	# MD	# VMD	% EA	% CA
Ampicillin	6	5	1	0	6	6	0	0	0	100.0	100.0
Cefazolin	10	10	0	0	9	10	0	0	0	90.0	100.0
Cefoxitin	13	6	7	0	13	13	0	0	0	100.0	100.0
Ceftriaxone	23	11	12	0	21	22	0	1	0	91.3	95.7
Ceftazidime	23	9	14	0	22	23	0	0	0	95.7	100.0
Cefepime	23	5	16	2	20	18	5	0	0	87.0	78.3
Amoxicillin-clavulanic acid	13	6	6	1	13	13	0	0	0	100.0	100.0
Piperacillin-tazobactam	9	3	5	1	8	8	1	0	0	88.9	88.9
Meropenem	21	6	15	0	20	21	0	0	0	95.2	100.0
Gentamicin	23	2	20	1	23	20	3	0	0	100.0	87.0
Tobramycin	23	3	18	2	23	22	1	0	0	100.0	95.7
Amikacin	23	1	22	0	22	23	0	0	0	95.7	100.0
Ciprofloxacin	23	10	13	0	23	22	1	0	0	100.0	95.7
Levofloxacin	23	10	12	1	23	22	1	0	0	100.0	95.7
Tetracycline	23	7	15	1	23	22	1	0	0	100.0	95.7
Trimethoprim-sulfamethoxazole	17	6	11	0	17	17	0	0	0	100.0	100.0
Total	296	100	187	9	286	282	13 (4.4%)	1 (0.5%)	0 (0%)	96.6	95.3

Results for 23 Gram-negative isolates comprising seven species, *E. coli* (6), *E. cloacae* (2), *K. pneumoniae* (3), *K. oxytoca* (4), *C. freundii* (2), *S. marcescens* (4), and *K. aerogenes* (2). The total number of isolates tested for each antimicrobial, along with number of resistant (R), susceptible (S), and intermediate (I) isolates is shown. The number of isolates tested in categorical agreement (CA) and essential agreement (EA) is displayed with the total percentage EA and CA and percentage rates of minor (mD), major (MD), and very major discrepancies (VMD).

**Table 2:** Disk diffusion antimicrobial susceptibility results for the eMcF compared to the traditional 0.5 McF.

Antibiotic (Disk)	# isolates	# R	# S	# I	# in CA	# mD	# MD	# VMD	%CA
Ampicillin	6	5	1	0	6	0	0	0	100.0
Cefazolin	13	11	2	0	13	0	0	0	100.0
Cefoxitin	13	5	7	1	13	0	0	0	100.0
Ceftriaxone	23	11	11	1	22	1	0	0	95.7
Cefepime	23	7	13	3	21	2	0	0	91.3
Amoxicillin-clavulanic acid	13	7	6	0	13	0	0	0	100.0
Piperacillin-tazobactam	18	3	14	1	18	0	0	0	100.0
Meropenem	23	5	16	2	21	1	1	0	91.3
Ertapenem	23	5	14	4	19	4	0	0	82.6
Gentamicin	23	2	20	1	22	1	0	0	95.7
Tobramycin	23	3	19	1	21	2	0	0	91.3
Levofloxacin	23	11	11	1	23	0	0	0	100.0
Total	224	75	134	15	212	11 (4.9%)	1 (0.7%)	0 (0%)	94.6

Results for 23 Gram-negative isolates comprising seven species, *E. coli* (6), *E. cloacae* (2), *K. pneumoniae* (3), *K. oxytoca* (4), *C. freundii* (2), *S. marcescens* (4), and *K. aerogenes* (2). The total number of isolates tested for each antimicrobial, along with number of resistant (R), susceptible (S), and intermediate (I) isolates is shown. The number of isolates tested in categorical agreement (CA) is displayed with the total percentage CA and percentage rates of minor (mD), major (MD), and very major discrepancies (VMD).

Of the 13 mDs observed on the VITEK2, five occurred in cefepime, three in gentamicin and one each in piperacillin-tazobactam, ciprofloxacin, levofloxacin, tetracycline, and tobramycin. The results of 16 antibiotics on the VITEK2 GN81 card were evaluated and all scored ≥ 90% in EA and CA, except cefepime which scored 87% and 78% respectively, and piperacillin-tazobactam which scored 88% for both EA and CA. Of the 11 mDs in disk diffusion, four occurred in ertapenem, two each in cefepime and tobramycin and one each in ceftriaxone, gentamicin, and meropenem. All 12 antibiotics tested by disk scored ≥ 90% CA, except ertapenem which scored 82%.

One MD was observed on the VITEK2 (ceftriaxone) and one in disk diffusion (meropenem) and both occurred in a single isolate (*K. oxytoca* CDC-147) which harbors the KPC-3 gene. Ceftriaxone tested susceptible (minimum inhibitory concentration (MIC) = 1 µg/ml) using the control 0.5 McF inoculum and resistant (MIC = 4 µg/ml) using the eMcF. Similarly, meropenem tested susceptible on disk diffusion (zone size of 23 mm) using the control 0.5 McF, and resistant (zone size of 19 mm) using the eMcF. The CDC reports both interpretations as Intermediate (MIC = 2). The control plate count was 1.03 × 10^8^ CFU/ml and the eMcF plate count was 1.86 × 10^8^ CFU/ml—both of which fall within the appropriate range for a 0.5 McF (1–2 × 10^8^ CFU/ml). These discrepancies are therefore likely to be particular to the isolate tested rather than the eQUANT method employed to generate the AST inoculum. The MICs and interpretations of CRE (carbapenem resistant Enterobacterales) strains have been reported to vary from day to day on the VITEK2^28^, and more recently it was demonstrated that the inoculum effect manifested in CRE strains, even within the CLSI recommended inoculum range, which served as a source of error and inconsistency in AST determinations for antibiotics such as meropenem and cefepime^29^.

For the acceptable performance of an antimicrobial susceptibility test, the FDA recommends the categorical agreement should be ≥ 90%, with mDs ≤ 10%, MDs ≤ 3% and VMDs ≤ 1.5%.

The combined AST results presented here satisfy these recommended performance guidelines and demonstrate that using the eMcF to replace the 0.5 McF as the AST inoculum could be a viable approach for the clinical laboratory.

### Performance of the eQUANT method using positive blood cultures up to twelve hours post flag time

**Figure 5:**
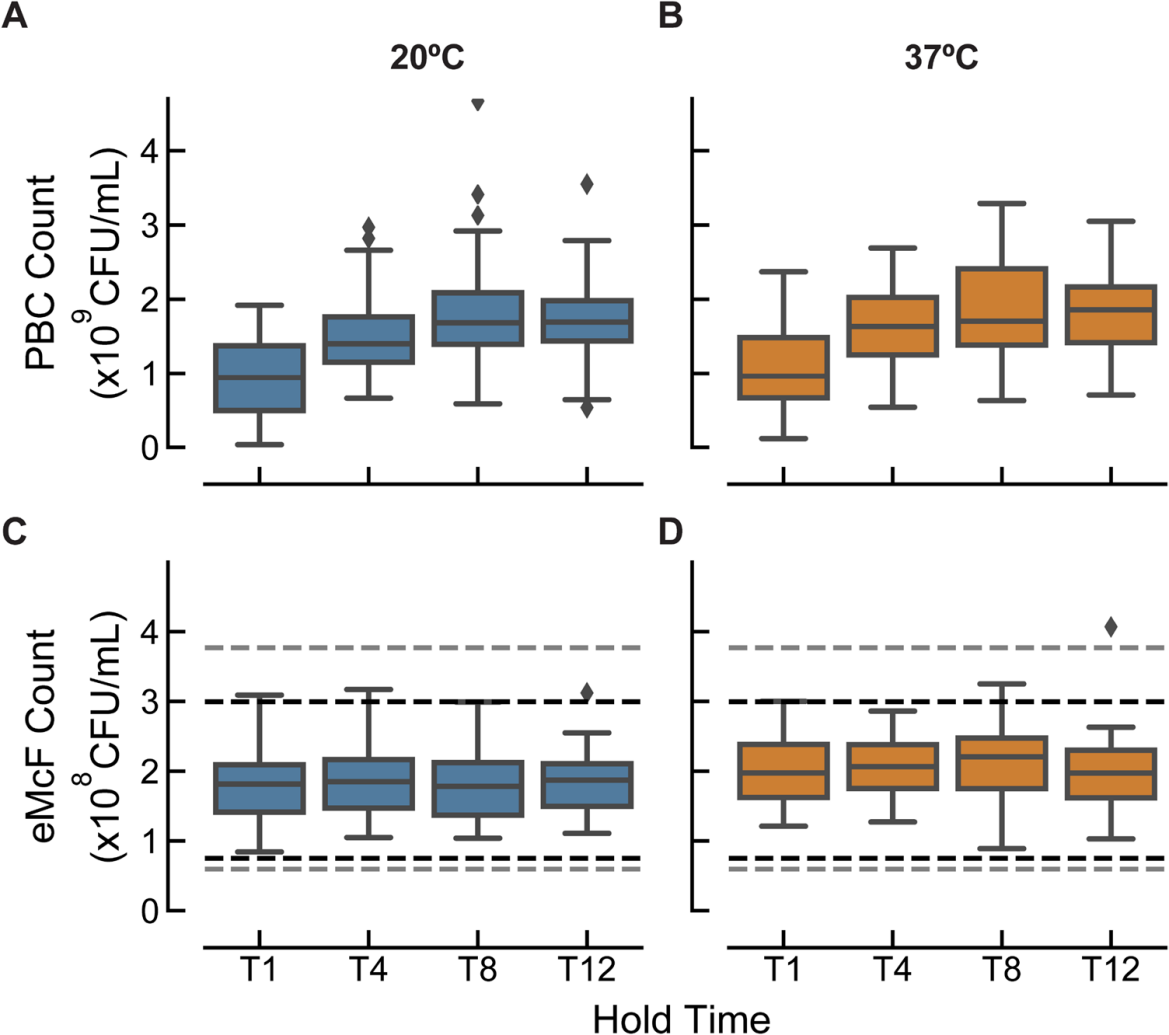
eMcF generation from PBCs at different time points post flag time. Aerobic and anaerobic blood culture bottles were spiked in duplicate with human blood and clinically significant Gram-negative isolates and incubated to positivity. One set of bottles was then removed from the blood culture instrument, incubated further at room temperature (20°C), and aliquoted at 1, 4, 8, and 12 h. The other set was incubated further on the blood culture system at 37°C and aliquoted at 1, 4, 8, and 12 h. (**A**, **B**) At each time point, the bacterial concentration in the PBC was determined by plate counts (20°C in blue, 37°C in orange). PBC counts covered a fivefold range for both temperatures. (**C**, **D**) Resulting eMcFs were plated for colony counts to assess the accuracy of the eQUANT method using PBCs held post flag time for up to 12 h at 20°C (blue) or 37°C (orange). Dotted lines represent the log10 ranges around the target 1.5 × 10^8^ CFU/mL (0.3 log, 0.4log).

We evaluated 208 eMcFs which were generated from PBCs sampled at two different temperatures and at four different timepoints. Aerobic and anaerobic blood culture bottles were inoculated with strains of 9 clinically significant Gram-negative aerobic organisms (*E. coli*, *K. pneumoniae*, *K. oxytoca*, *E. cloacae*, *K. aerogenes*, *S. marcescens*, *C. freundii*, *P. mirabilis*, *P. vulgaris*) and incubated to positivity. These PBCs were then held at either 20 °C (on the bench at room temperature) or 37°C (on the blood culture instrument itself). The time points tested were one (T1), four (T4), eight (T8), and 12 (T12) hours post flag time. Each PBC sample was diluted 1:30 into growth media and the benchtop-equivalent eQUANT setup was used to measure ORP. Algorithms used for eMcF determination were unchanged, and the resulting eMcFs, 128 eMcFs from aerobic PBCs and 80 eMcFs from anaerobic PBCs, were plated for colony counts (Fig. 5C, D).

As expected, PBC bacterial concentrations were lowest when measured at T1 and rose significantly over time, reaching approximate saturation at T8-T12 (Fig. 5A, B). Despite the extended incubation both on and off the blood culture instrument (which allowed the bacterial concentration to increase significantly), 96% of the eMcF results were within ± 0.3log, and 78% were within ± 0.2log of the target 1.5 × 10^8^ CFU/ml.

## Discussion

Rapid antimicrobial therapy is critical for patients with a bloodstream infection, and the suitability of the initial empiric antibiotics administered by the clinician must be confirmed by AST results as soon as possible. Most clinical laboratories utilize automated AST platforms, the starting inocula of which tend to rely on 0.5 McFs prepared from the colonies of a PBC subculture. Typical timelines for this subculturing step can exceed 18 h, and manual preparation of the 0.5 McF is still required. Using the innovative eQUANT method developed and presented here, equivalent eMcFs were generated in 1–2 h post-rapid ID offering potentially significant time savings.

The eQUANT leverages novel electrical sensor technology and organism-specific algorithms to quantify bacterial growth in a diluted positive blood culture sample. The proof-of-concept study described here demonstrates that the method accurately generates a standardized inoculum, the eMcF, and further demonstrates the excellent performance of the eMcFs for several clinically significant Gram-negative organisms on both a manual and automated AST platform. A majority of our eMcFs were completed within our initial target range of 1–2 h (average of 106 ± 28 min), with the longest eMcF taking 2.5 h. Additionally, this time saving is achieved without sacrificing any accuracy for the bacterial concentration of our eMcF compared to a traditional 0.5 McF, with 91% of the eMcFs within ± 0.3log of the target 1.5 × 10^8^ CFU/ml.

Automation of laboratory processes offers key benefits such as saving time, avoiding human errors, and improving workflow in the clinical lab. The eQUANT easily automates the preparation of a standardized AST inoculum from a PBC. As the eMcF is equivalent to a traditional 0.5 McF, it is not restricted to a single AST platform and can potentially be used in any downstream AST process which currently relies on a 0.5 McF as the starting inoculum, including automated AST systems. Polymicrobial PBC samples remain a limitation and a challenge for the eQUANT technology as indeed for any currently available rapid ID or AST system^30,31^. Currently, only PBCs confirmed monomicrobial via Gram staining and rapid ID are processed on the eQUANT and the presence of a high white blood cell count does not affect eMcF generation (see Supplementary Information Figure S5).

For clinical laboratories that may conduct batch processing of PBCs, it is critical that the further incubation of positive blood cultures (either on the blood culture instrument or on the benchtop) does not affect the eQUANT method. We therefore investigated the accuracy of eMcF generation from positive blood cultures at 1, 2, 4, and 12 h post flag time and demonstrated that the eMcF is agnostic to time holds up to 12 h, both on the bench and on the blood culture instrument itself. Despite PBC concentrations at the various time points ranging from 3.6 × 10^7^ to 4.7 × 10^9^ CFU/ml, 96% of all eMcF counts fell within ± 0.3log of the target 1.5 × 10^8^ CFU/ml. This ensures the eQUANT can accommodate a variety of potential laboratory workflows.

The proof-of-concept study results presented here are limited in both the scope of the organisms tested and the relatively small sample size. We have continued to improve and expand our technology, specifically by generating algorithms to include further clinically significant organisms such as *P. aeruginosa* and *A. baumannii* along with species of *Staphylococci* and *Enterococci*. Studies on these organisms are ongoing. Future research will focus on further evaluating organisms on the eQUANT device, investigating the application of the eQUANT technology to clinical specimens other than PBCs, and examining the role of the eQUANT technology in fully automated clinical microbiology workflows.

## Methods

### Sensor fabrication

The disposable, one-time use ORP sensor has been developed in collaboration with Analog Devices International (ADI) using microfabrication techniques and open cavity integrated circuit (IC) packaging. The sensor consists of a lithographically patterned platinum electrode fabricated on a silicon/silicon oxide substrate in a cleanroom environment. The Si substrate is diced into individual dies and packaged by wire bonding to a 5 mm x 5 mm printed circuit board (providing connections to the readout electronics) followed by film assisted molding (FAM) of an open cavity package which covers the wire bonds and bond pads and exposes only the platinum electrode sensor to the liquid sample.

### Sensor integrated eTube cartridge

The disposable sensor cartridge (eTube) is made using polycarbonate (PC) plastic injection molding and the FAM packaged sensor is inserted into and attached to a fitted eTube opening using a UV-cure adhesive (Dymax MD 1405 M-T-UR-SC). The reference electrode is a silver/silver chloride electrode built into the cap of the cartridge which provides a stable potential during the measurement. The eTubes were cleaned with 99.5% IPA with sonication for 10 min to prevent environmental contamination, and each eMcF was plated for colony counts which served as an additional purity check.

### eQUANT instrument and workflow

The eQUANT instrument consists of a heater module that holds one eTube and maintains the temperature at 37 °C during sample run and incubation. Once the run is completed and the target bacterial concentration (0.5 McF) has been reached, it cools the sample to 15 °C. The readout electronics include high input impedance op-amps and analog-to-digital converters, with a microcontroller processing the sensor signal in real-time and applying the algorithms pre-loaded into the firmware. The software and graphical user interface allow for organism ID selection and algorithm integration.

During an experimental run, PBCs were diluted 1:30 in cation-adjusted Mueller Hinton growth medium (Hardy Diagnostics) and 1 mL was transferred into the eTube and placed into the eQUANT instrument which incubated the sample at 37 °C for the duration of organism growth. Once the eMcF was reached (as determined by the eQUANT algorithms, see Supplementary Information, Figure S1–S3), the sample was removed from the instrument, plated for colony counting as described above, and used for downstream AST.

### Benchtop setup for eQUANT algorithm generation

In order to increase experimental throughput during instrument development and prototyping, we developed an equivalent benchtop setup using commercially available ORP electrodes (Mettler Toledo InLab Redox Micro) which exhibit nearly identical ORP response to the sensors used in the eQUANT. Instead of an eTube cartridge, samples were contained in conical tubes, and heating for incubation was provided by a dry bath heating block. The ORP probe was inserted into the tube for real-time signal monitoring, and the voltage was read out using an ORP measurement board from Atlas Scientific (EZO ORP) which provided a serial interface. Measurements were recorded from the serial output via a Python script. This setup was used extensively in the algorithm development process as it allows for simultaneous monitoring of ORP signal and bacterial concentration for each sample.

### Isolates

A total of 23 Gram-negative test isolates were obtained from the following sources: Clinical Microbiology Laboratory at Stanford Health Care (2 *E. coli*, 1 *K. pneumoniae*), Clinical Microbiology Laboratory at UCLA (4 *E. coli*, 2 *K. oxytoca*, 1 *K. aerogenes*, 1 *E. cloacae*, 2 *C. freundii*, 2 *S. marcescens*), CDC AR Bank (*K. oxytoca* CDC 147, *K. oxytoca* CDC 837, *E. cloacae* CDC 644, *S. marcescens* CDC 124, *K. aerogenes* CDC 547, *K. pneumoniae* CDC 5), BEI (*K. pneumoniae* NR 48563), and ATCC (*S. marcescens* ATCC 14756). Information regarding the molecular mechanisms of resistance (MMR) was available for four of the CDC AR Bank isolates; KPn CDC 5 (KPC-2), KOx CDC 147 (KPC-3), KOx CDC 837 (IMP-4), SMa CDC 124 (SME-3). MMR data was unavailable for the remaining isolates. All isolates were frozen, stored at -80 °C in glycerol stock and subcultured twice on blood agar before use. Plates were incubated in air at 35 °C for 18–24 h.

### Sample preparation

BD BACTEC Plus Aerobic Blood Culture Bottles (Fisher Scientific) were inoculated with 9 mL of human whole blood (with sodium citrate as the anti-coagulant) purchased from Lampire Biological Laboratories Inc. (Catalog No. 7203706). The blood was tested at Lampire using their standard quality control process. A 0.5 McF of the test organism was prepared from colonies and diluted before spiking the blood culture bottle to produce a final concentration of 2–3 CFU/mL. Bottles were incubated in the BD BACTEC 9050 System until flagged as positive by the instrument. PBCs were then diluted in saline (Hardy Diagnostics) and plated on blood agar for colony counts before the eQUANT method was performed. The drop count method was used, in which seven 10 uL drops were evenly placed onto a blood agar plate which was incubated in air at 35 °C for 18–24 h. Following incubation, the number of colonies in each drop was counted and the dilution factor was applied to calculate the CFU/ml concentration of the PBC.

### Evaluating the use of PBCs up to 12 h post flag time

BD BACTEC Plus Aerobic and Anaerobic blood culture bottles (Fisher Scientific) were spiked with human blood as described above and inoculated with 2–3 CFU/ml of a range of clinically significant Gram-negative aerobic isolates. Aerobic blood culture bottles were inoculated with *E. coli* (2), *K. pneumoniae* (2), *K. oxytoca* (2), *E. cloacae* (2), *K. aerogenes* (2), *S. marcescens* (2), *C. freundii* (2), *P. mirabilis* (2), and *P. vulgaris* (1). Anaerobic blood culture bottles were inoculated with *E. coli* (2) *K. pneumoniae* (1), *K. oxytoca* (1), *E. cloacae* (1), *K. aerogenes* (1), *S. marcescens* (1), *C. freundii* (1), *P. mirabilis* (1), and *P. vulgaris* (1). Isolates (n = 18) were obtained from the following sources: Clinical Microbiology Laboratory at Stanford Health Care (7), Clinical Microbiology Laboratory at UCLA (8), CDC AR Bank (2) and ATCC (1). For each bottle/isolate combination, two blood culture bottles were inoculated and incubated in the BD BACTEC 9050 System to positivity. At flag time, one PBC was removed and incubated further on the bench at room temperature (RT) with the removal and testing of aliquots after one (T1), four (T4), eight (T8) and 12 h (T12). The other PBC was further incubated on the blood culture instrument at 37 °C, and aliquots were also removed at T1, T4, T8 and T12. The aliquots were immediately diluted and plated for colony counting as previously described and eMcFs (n = 208) were generated using the eQUANT method on the equivalent benchtop setup.

### Antimicrobial susceptibility testing using eMcF compared to conventional 0.5 McF

AST was performed using the eMcF as the test inoculum and a traditional 0.5 McF, prepared from an overnight subculture, as the control inoculum. Antimicrobials were selected for testing based on their inclusion in the CLSI Group A and B reporting categories for *Enterobacterales*^14^.

### Kirby–Bauer disk diffusion

The control 0.5 McF and the test eMcF inocula were lawned onto 15 × 150 mm Mueller Hinton agar plates (Hardy Diagnostics) in accordance with CLSI recommendations^15^, and 12 antimicrobial infused disks were applied to the plate using a BD BBL™ Sensi-disk™ dispenser. The following antimicrobial disks were tested: piperacillin-tazobactam (100/10 µg), (10 µg), levofloxacin (5 µg) and amoxicillin-clavulanic acid (20/10 µg). Disks were supplied by Hardy Diagnostics except cefepime, ertapenem and meropenem which were supplied by BD. Five of the 23 isolates (3 *K. oxytoca*, 2 *K. pneumoniae*) were not tested with piperacillin-tazobactam on disk diffusion due to disc shortage at the time of testing. Quality control was carried out according to the manufacturer’s instructions.

### VITEK2 AST

The control 0.5 McF and the test eMcF inocula were manually diluted according to the manufacturer’s instructions and used to inoculate AST-GN81 cards^32^. Purity plates were made from the remaining suspension in the AST dilution tube following processing. The following antibiotics were evaluated from the AST-GN81 card: meropenem, ampicillin, cefepime, amoxicillin-clavulanic acid, cefoxitin, ceftriaxone, tobramycin, gentamicin, piperacillin-tazobactam, levofloxacin, cefazolin, ceftazidime, amikacin, tetracycline, trimethoprim-sulfamethoxazole, and ciprofloxacin. The VITEK2 GN81 results for piperacillin-tazobactam for *S. marcescens* were suppressed, and MIC results for the following drug/organism combinations were excluded from analysis as they are not indicated for use by the FDA according to the GN81 package insert: meropenem with *K. aerogenes;* piperacillin-tazobactam with *K. oxytoca*, *K. aerogenes*, *E. cloacae*, *S. marcescens*, *C. freundii*; trimethoprim-sulfamethoxazole with *C. freundii* and *S. marcescens.* Quality control was carried out according to the manufacturer’s instructions.

### Antimicrobial susceptibility data analysis

Essential agreement, categorical agreement, very major discrepancies, major discrepancies, and minor discrepancies were defined and calculated in the following manner. Essential agreement (EA) is defined as the percent of all eMcF MIC results within one twofold dilution of the control MIC result. Categorical agreement (CA) is the percent of all eMcF results with the same category interpretation as the reference disk diffusion and comparator VITEK2 result. A minor discrepancy (mD) was defined as an intermediate (I) result using the eMcF method compared to a susceptible (S) or resistant (R) result using the control method, or (I) by the control method and (S) or (R) by the eMcF method. A major discrepancy (MD) was defined as (R) using the eMcF method and (S) using the control method, and a very major discrepancy (VMD) was defined as (S) using the eMcF method and (R) using the control method.

The MD rate was calculated by dividing the number of discrepancies by the number of isolates which tested susceptible using the control method. The VMD rate was calculated by dividing the number of discrepancies by the number of isolates which tested resistant using the control method^33^.

The MIC and the zone diameter results, generated by the VITEK2 and disk diffusion respectively, were interpreted using CLSI M100 29^th^ edition breakpoints^14^ for *Enterobacterales*, except for (1) Ciprofloxacin and Levofloxacin for which CLSI M100 28^th^ edition breakpoints^15^ were used, and (2) Cefazolin and Cefoxitin for which current FDA breakpoints^34^ were used. Antibiotic results for isolates with intrinsic resistance^14^ were excluded from analysis.

### Figures and Images

Plots were generated with Python 3.0 (www.python.org), using the matplotlib (matplotlib.org) and seaborn (seaborn.pydata.org) libraries. Schematics were created using BioRender (www.biorender.com). Photos were taken in house and used with the permission of the owners.

## Supplementary information

Supplementary Information.

## Data Availability

The authors declare that the data generated and analyzed during this study are available within the article and the Supplementary Information file.
