# Supplementary material for: Novel electronic biosensor for automated inoculum preparation to accelerate antimicrobial susceptibility testing
Source: Sci Rep. 2021 May 31;11:11360. doi: 10.1038/s41598-021-90830-2 (PMC8166823; doi:10.1038/s41598-021-90830-2)
Supplement: Supplementary file 1 — Supplementary Information. [file 41598_2021_90830_MOESM1_ESM.docx]

**Supplementary Information**

**Title:** Novel electronic biosensor for automated inoculum preparation to accelerate antimicrobial susceptibility testing

**Authors:** Suzanne Putney, Andy H. Theiss, Nitin K. Rajan, Eszter Deak, Creighton Buie, Yvonne Ngo, Hima Shah, Victoria Yuan, Elizabeth Botbol-Ponte, Adrian Hoyos-Urias, Oren Knopfmacher, Catherine A. Hogan, Niaz Banaei, Meike Herget

**eQUANT Algorithm Type 1 (Single Threshold)**

The first type of algorithm used by the eQUANT system to produce an eMcFarland is a single threshold algorithm. For this algorithm type, the instrument monitors the real-time ORP signal to determine when a threshold change in voltage value (ΔORP) is reached. This threshold value is species-specific and provided in the lookup table (LUT). Once this threshold is achieved, the growth is complete and the eMcFarland sample is ready.

An example of a species that currently utilizes a Type 1 algorithm is *Serratia marcescens*. The LUT for *S. marcescens* specifies that the target eMcFarland concentration will be reached once ΔORP = 280mV. This delta is calculated from the ORP value read by the sensor at a designated normalization time (e.g. t_norm_ = 10 min). Thus, the instrument compares the current sensor readings to the normalization value to determine when a delta of 280mV has been reached.

Figure S1 below shows an example of the application of a Type 1 algorithm to a real-time *S. marcescens* ORP signal. The normalization value in this case was V_norm_ = 0.192 V, and a ΔORP of 280mV gives a target eMcFarland threshold of V_thresh_ = -0.088 V. The instrument monitors the real-time signal until this threshold is reached, and then cools the sample and notifies the user that the eMcFarland sample is ready for AST. In the example ORP curve below, the algorithm determined the run was completed after 96.5 minutes, and the eMcFarland concentration was 1.87e8 CFU/ml.


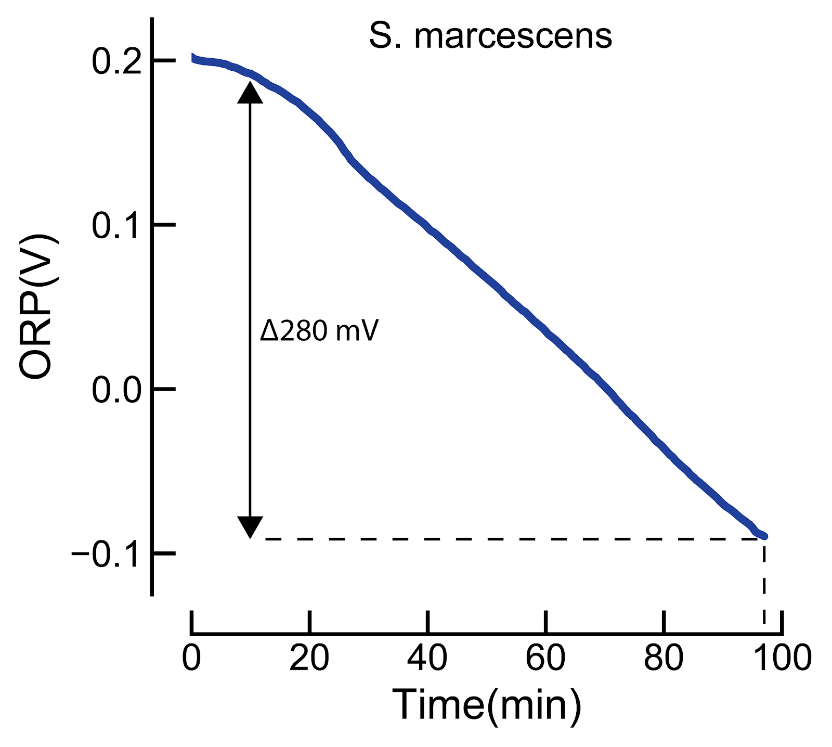


**Figure S1. Example of eQUANT Type 1 algorithm applied to *S. marcescens* ORP signal.** The real-time ORP signal of *S. marcescens* is measured every 10 seconds to determine when a threshold ΔORP of 280mV is reached (dashed blue line). This delta is calculated by comparing the current ORP sensor reading to the normalization value measured at 10 minutes into the run. Once this threshold is reached (red dashed line), the run is completed and the eMcFarland sample is ready.

**eQUANT Algorithm Type 2 (Single Threshold + Time)**

The second type of algorithm used by the eQUANT system to produce an eMcFarland is a single threshold plus time algorithm. This algorithm type monitors the real-time ORP signal to determine when a threshold ΔORP value is reached and then adds a specified time. Once this time has elapsed, the eMcFarland sample is ready.

An example of a species that currently utilizes a Type 2 algorithm is *Klebsiella pneumoniae*. The LUT for *K. pneumoniae* specifies a threshold of ΔORP = 150mV and added time of Δt = 40 minutes. Thus, the eQUANT instrument compares the current sensor readings to the normalization value to determine when a delta of 150mV has been reached, continues sample growth for an additional 40 minutes, and then notifies the user that the eMcFarland is ready.

Figure S2 below shows an example of the application of a Type 2 algorithm to a real-time *K. pneumoniae* ORP signal. The normalization value in this case was V_norm_ = 0.192 V, giving a target eMcFarland threshold value of V_thresh_ = 0.042 V. The instrument monitors the real-time signal until this threshold is reached, continues growth for 40 minutes, and then cools the sample and notifies the user that the eMcFarland sample is ready. In the example ORP curve below, the threshold was reached at 61.5 minutes, the run completed at 101.5 minutes, and the eMcFarland concentration was 1.56e8 CFU/ml.


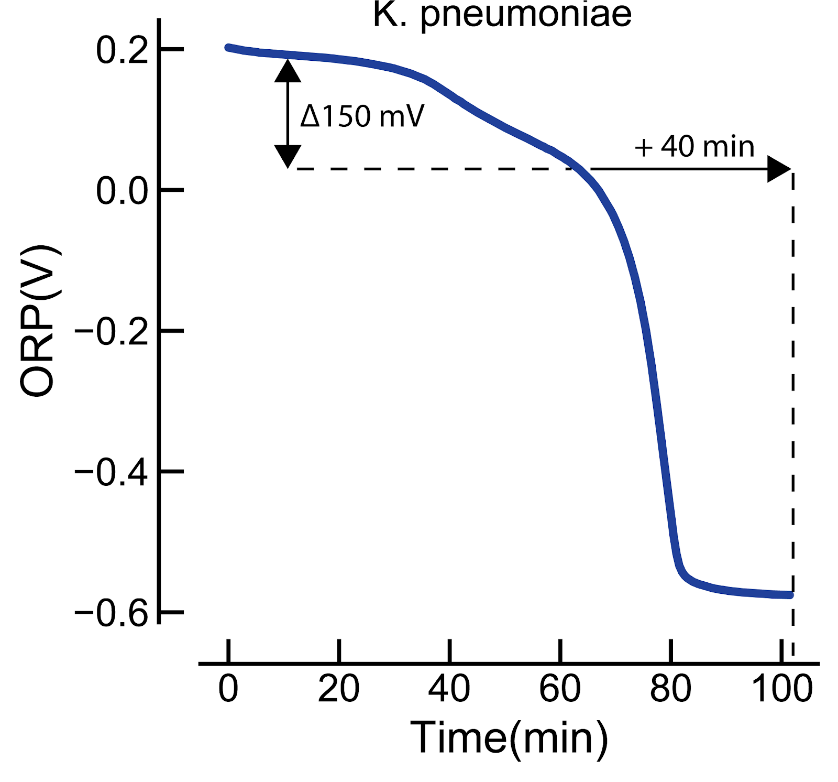


**Figure S2. Example of eQUANT Type 2 algorithm applied to *K. pneumoniae* ORP signal.** The lookup table for *K. pneumoniae* specifies a threshold of Δ150mV and a time delta of 40 minutes. The real-time ORP signal is measured every 10 seconds to determine when this threshold is reached (dashed blue line). A delta of 40 minutes is then added to the threshold time to determine when the growth will be completed and the target eMcFarland has been reached (red dashed line).

**eQUANT Algorithm Type 3 (Two-Threshold Prediction)**

The third type of algorithm used by the eQUANT system to produce an eMcFarland utilizes two thresholds and a scaling factor. This algorithm type monitors the real-time ORP signal to determine the time points at which the two threshold values are reached, and then uses the time difference between these points as well as a scaling factor to determine the time when the eMcFarland growth is complete.

For a Type 3 algorithm, the species-specific LUT provides three parameters - the two threshold delta values (*ΔORP1*, *ΔORP2*) and a scaling factor (*s*). The time points at which these thresholds are reached (*t_1_*, *t_2_*) are then used to calculate the time for a completed eMcFarland (*t_emf_*). The equation for this calculation is shown below by Equation S1:

$t_{emf}=t_{2}+\left( t_{2}-t_{1} \right) \times s$ (Equation S1)

An example of a species that currently utilizes a Type 3 algorithm is *Escherichia coli*. The LUT for *E. coli* specifies the following algorithm values: *ΔORP1 =* 200mV, *ΔORP2 =* 400mV, and *s* = 2.96. Thus, the instrument compares the current sensor readings to the normalization value to determine the time points when the thresholds are reached, and then calculates the time to a completed eMcFarland using Equation S1.

**Figure S3** below shows an example of the application of a Type 3 algorithm to a real-time *E. coli* ORP signal. The normalization value in this case was V_norm_ = 0.223 V, giving target threshold values of 0.023 V and -0.177 V. The instrument monitors the real-time signal until these thresholds are reached, and then calculates the completion time using these threshold time points. The calculation for the time at which the eMcFarland sample will be ready is shown below:

t_1_=61.5 min

t_2_=66.5 min

t_emf_ = 66.5 + (66.5-61.5) x 2.96 = 81.3 min

In the example ORP curve below, the run completed at 81.5 minutes and the eMcFarland concentration was 1.99e8 CFU/ml.


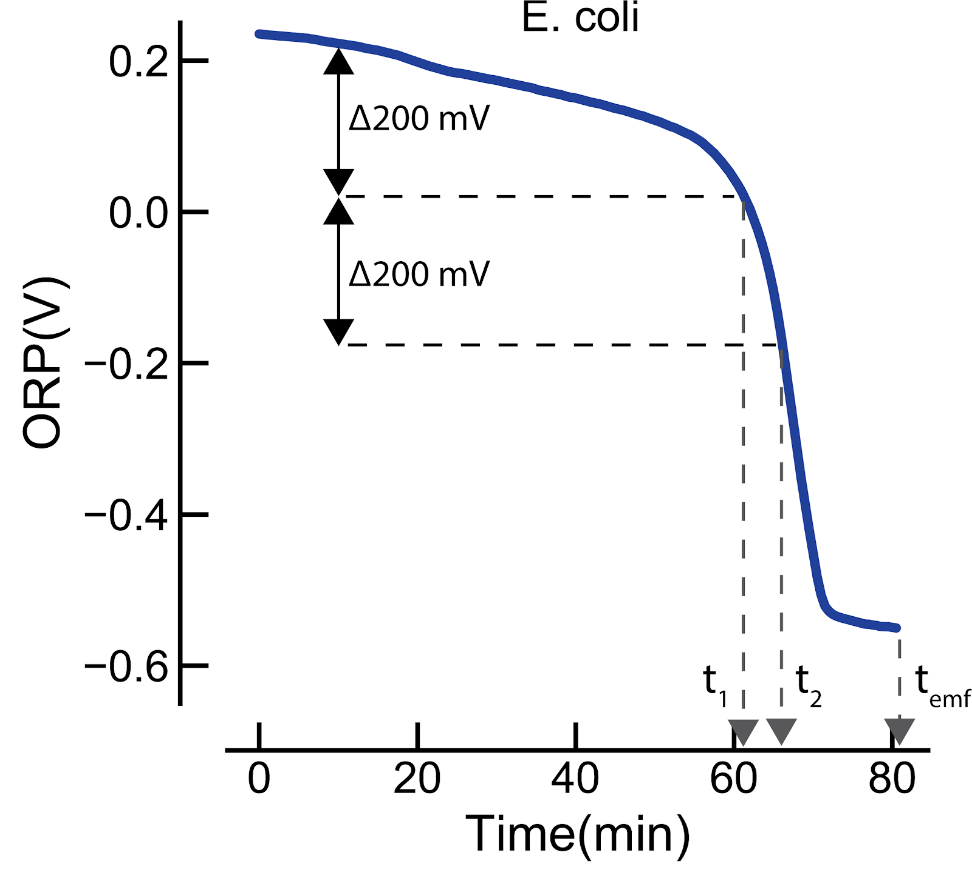


**Figure S3. Example of eQUANT Type 3 algorithm applied to *E. coli* ORP signal.** The eQUANT LUT for *E. coli* specifies thresholds of Δ200mV and Δ400mV and a scaling factor of 2.96. The real-time ORP signal is measured every 10 seconds to determine when the thresholds are reached (Δ200mV shown by dashed blue line, Δ400mV shown by dashed green line). Equation S1 is then used to calculate when the growth will be completed and the target eMcFarland has been reached (red dashed line).

**Original ORP Curves (from Figure 3B) -- Capturing Strain Variation**

The ORP signals depicted in the Results section (Figure 3B) were shifted in time to align the curves and illustrate that, despite measuring varied strains from a bacterial species, there are significant similarities in the ORP curve during growth that can be utilized for algorithm development and real-time application. However, it is also important to point out that in real-time growth there are often slight differences in the timing and minor features of the ORP curve. These differences arise from changes in the starting inoculum concentration as well as the individual strain growth rates.

Figure S4 below shows the differences in growth rates for five strains across six species. The starting inocula were prepared to be of equivalent concentration (1x10^7^ CFU/mL), so the variations seen in the ORP signals are primarily attributed to strain to strain growth differences. While growth rates for various strains within a species are often similar, they are not identical and encompass a distribution of growth rates. Thus, it is necessary to use a collection of strains in the algorithm development process in order to capture a representative distribution of growth characteristics for the species.

**
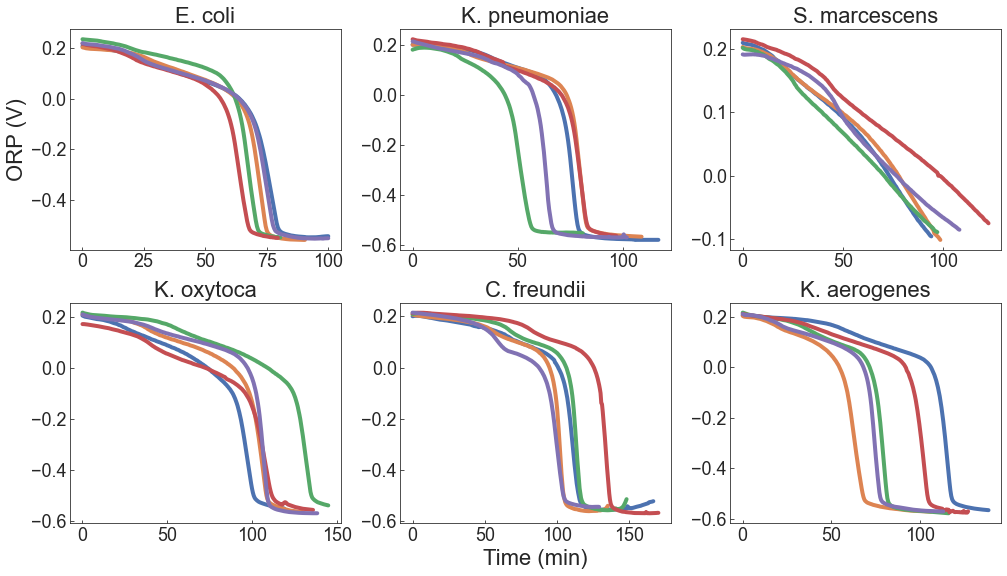
**

**Figure S4. Original ORP signals from multiple bacterial species.** Five different strains of six organisms (*E. coli*, *K. pneumoniae*, *S. marcescens*, *K. oxytoca*, *C. freundii*, *K. aerogenes*) were cultured and bacterial suspensions of ~1x10^7^ CFU/mL were prepared. These were grown at 37°C and the ORP curves are shown here. Variations in the timing and minor differences in the ORP curve shapes are largely attributed to variances in growth characteristics between the individual strains.

**Investigating the effect of positive blood cultures containing a high white blood cell concentration on the eQUANT algorithm and eMcF generation**

Patients presenting with a bloodstream infection may also have a high white blood cell count. This warrants an investigation of white blood cells as a potential interfering substance present in a positive blood culture. The normal range of white blood cells is 4000-11,000 per ul/whole blood. We tested the eQUANT using samples spiked to simulate PBCs from patients with a high WBC count ( $\geq$12,000 WBCs/ul).

Briefly, blood cultures were spiked with *E. coli* and *A. baumanii*, incubated until positivity and run on eQUANT as control samples. These PBCs were also diluted into duplicate test eTubes which were then spiked with a concentration of white blood cells to simulate a positive blood culture from a patient with >=12,000 WBCs/ul before being run on eQUANT.

The white blood cells were derived from a “Buffy Coat” suitable for our research purposes (prepared from a same day blood draw using a single donor) which was purchased from Stanford Blood Center. It was diluted 1:100 in PBS and the white blood cells were enumerated using a Neubauer Haemocytometer. The Buffy Coat was then used immediately to spike the eQUANT samples with the required number of white blood cells.

As Figure S5 below shows, the duplicate test runs (labelled Buffy Coat 1 and Buffy Coat 2) are comparable to the control runs both in signal shape and time to eMcF. Further, the eMcF test plate counts were within 0.2log of the eMcF control plate counts, indicating that a PBC from a patient with a WBC count >=12,000/ul does not significantly affect the generation of the eMcF.


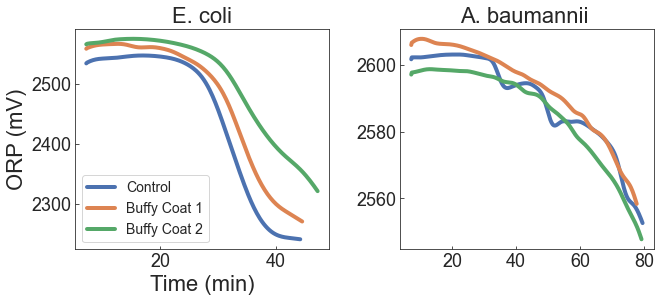


**Figure S5.** ORP signals from PBCs containing *E. coli* and *A. baumanii* which were spiked with white blood cells (12,000 WBCs/ul whole blood). The duplicate test runs (labelled Buffy Coat 1 and Buffy Coat 2) are comparable to the control runs both in signal shape and time to eMcF.
